# Immunopathogenesis of antibody-mediated rejection in liver transplantation

**DOI:** 10.3389/fimmu.2026.1838990

**Published:** 2026-06-03

**Authors:** Bo Luo, Mengyue Wang, Guoyu Chen, Wang Li, Yuanyi Mang, Shengning Zhang

**Affiliations:** 1 Department of Hepatobiliary and Liver Transplantation Surgery, The First People’s Hospital of Yunnan Province, Kunming, Yunnan, China; 2 Kunming Medical University, Kunming, Yunnan, China

**Keywords:** antibody-mediated rejection, complement, donor-specific antibody, liver transplantation, microvascular inflammation

## Abstract

Antibody-mediated rejection (AMR) after liver transplantation (LT) is increasingly recognized as a cause of graft dysfunction, chronic injury, and graft loss, but remains difficult to diagnose because serologic, histologic, and clinical findings are often incomplete or overlapping. This review focuses specifically on liver AMR and distinguishes liver-specific evidence from mechanisms inferred from experimental models or other solid-organ transplants. We summarize how donor-specific antibodies (DSA), complement activation, Fc receptor-mediated effector mechanisms, endothelial activation, and microvascular inflammation interact with the liver graft’s relative resistance to humoral injury. We also discuss the diagnostic overlap among acute AMR, chronic AMR, T cell-mediated rejection, and plasma cell-rich rejection, and propose a practical diagnostic-therapeutic framework integrating DSA, C4d, histology, graft dysfunction, and mechanism-based treatment options.

## 1 Introduction

Liver transplantation (LT) is an established treatment for end-stage liver disease. Advances in surgical technique, perioperative care, and immunosuppressive management have substantially improved post-transplant survival (1). Nevertheless, long-term graft outcomes remain limited by immune-mediated complications, among which antibody-mediated rejection (AMR) has received increasing attention. Although AMR is relatively uncommon after LT, it remains an important cause of graft dysfunction and graft loss (2).

The reported incidence of AMR after LT is considerably lower than that observed in kidney, heart, and lung transplantation (3, 4). This difference is generally attributed to the distinctive immunological properties of the liver allograft. The liver exists within a relatively tolerogenic immune environment, has a dual blood supply, and possesses a substantial capacity to absorb, neutralize, or clear circulating alloantibodies (5). In addition, the liver may release soluble human leukocyte antigen (HLA) class I molecules that bind donor-specific antibody (DSA), forming immune complexes and platelet aggregates that can subsequently be removed by Kupffer cells (KCs). Together, these features may partially protect the liver allograft from antibody-mediated injury (6).

This relative resistance, however, is not absolute. AMR still occurs after LT and has become an increasingly important clinical problem. When recognition is delayed or treatment is ineffective, AMR may progress to severe graft injury, graft failure, and even retransplantation (7). In practice, both diagnosis and management remain challenging. Clinical manifestations are often non-specific, histopathologic findings may overlap with those of other forms of graft injury, and treatment protocols vary substantially across transplant centers, with inconsistent therapeutic responses (4, 8). These difficulties likely reflect, at least in part, an incomplete understanding of the immunopathogenesis of liver AMR and the lack of clearly defined, mechanism-based treatment strategies.

AMR is a form of allograft injury that develops in the setting of circulating DSA or ABO blood group incompatibility (9). In kidney, heart, and lung transplantation, AMR is a well-recognized complication; in LT, by contrast, it has historically been considered less common and less clearly defined. Acute antibody-mediated rejection (aAMR) and chronic antibody-mediated rejection (cAMR) represent the two principal clinical manifestations of liver AMR, and they differ in timing, histopathologic features, and treatment response (3). Although the Banff Working Group has proposed a diagnostic framework for AMR in liver allografts, the mechanisms that drive its initiation, progression, and heterogeneity remain incompletely understood. Much of the current conceptual framework still derives either from limited liver-specific studies or from evidence extrapolated from other solid-organ transplants (9).

Current evidence indicates that liver AMR cannot be explained by antibody binding alone (10). Rather, it is a dynamic process involving DSA recognition of graft targets, complement activation, endothelial injury, recruitment of innate immune cells, and downstream inflammatory amplification. Unlike reviews that mainly summarize AMR across solid organs or provide a clinicopathologic update, this article is organized around a liver-specific question: why does a relatively resistant graft develop clinically meaningful humoral injury in only selected settings? Accordingly, this review distinguishes liver-specific evidence from extrapolated mechanisms, integrates cellular and pathway-level mechanisms with diagnostic and therapeutic implications, and identifies assumptions that still require direct validation in LT.

## 2 Relative resistance and residual susceptibility of the liver allograft to AMR

### 2.1 Mechanisms contributing to the relative resistance of the liver allograft

Compared with kidney, heart, and lung transplantation, AMR is less common after liver transplantation (3, 4), largely because the liver allograft has several protective anatomical and immunological features. These include a tolerogenic sinusoidal immune environment, dual blood supply, abundant resident macrophages, and the capacity to absorb, neutralize, or clear circulating DSA (5, 11). In addition, soluble human leukocyte antigen (HLA) class I molecules released by the liver graft may bind circulating DSA and promote the formation of immune complexes or platelet aggregates, which can subsequently be cleared by Kupffer cells (KCs) and other sinusoidal phagocytic cells (12). Together, these mechanisms may reduce the effective antibody burden at the graft interface and help explain the relative resistance of the liver allograft to overt antibody-mediated injury (12, 13).

### 2.2 Conditions that promote susceptibility to AMR

Despite these protective features, the liver allograft is not immune to antibody-mediated injury. The protective effect of the liver can be overcome when DSA is strong, persistent, newly developed after transplantation, directed against class II antigens, or capable of binding complement (14). Sustained exposure to such antibodies may promote complement activation, Fc receptor-mediated effector responses, endothelial activation, and microvascular inflammation.

The condition of the graft itself is also important. Ischemia-reperfusion injury, infection, biliary complications, inadequate immunosuppression, and overlapping T cell-mediated rejection (TCMR) can create a pro-inflammatory microenvironment in which endothelial cells are more easily activated, and leukocytes are more readily recruited. This may partly explain why some DSA-positive recipients remain clinically stable, whereas others develop graft dysfunction, chronic injury, or graft failure (15, 16). Therefore, AMR after LT is better viewed as the convergence of humoral alloimmunity and a permissive inflammatory graft environment, rather than as a direct consequence of antibody binding alone.

## 3 DSA biology in liver transplantation

Donor-specific antibody are antibodies in transplant recipients that recognize donor-derived human leukocyte antigen (HLA) class I, class II, or non-HLA antigens after organ or tissue transplantation (17). Among them, antibodies against HLA class II are detected more often. These antibodies are also more often linked to poor graft outcomes after solid organ transplantation (18, 19). Based on the time of appearance, DSA can be divided into preformed DSA and *de novo* DSA (dnDSA). Preformed DSA are usually found in previously sensitized recipients. This includes recipients with a history of multiple pregnancies, repeated blood transfusions, long-term hemodialysis, or viral infection (6, 12). In contrast, dnDSA usually develops after transplantation. It has been linked to insufficient immunosuppression, abnormal immune activation, and infection-related triggers (12). Current evidence suggests that preformed DSA is more often linked to early graft injury and acute rejection. In contrast, dnDSA seems to have a closer link with acute AMR, chronic AMR, and mixed rejection phenotypes after transplantation (20, 21).

Almost all hepatic cell types express HLA class I antigens. DSA against HLA class I tends to decrease over time and may disappear within about 12 months after liver transplantation (22). For this reason, these antibodies are usually considered more relevant to early post-transplant immune injury. In contrast, anti-HLA class II DSA tends to persist. These antibodies have been linked more consistently to long-term graft dysfunction and poor graft survival (23). This difference may reflect the different biological behavior of class II alloimmunity in the liver graft and its closer link with ongoing humoral immune activation.

Anti-HLA class II DSA may contribute more directly to endothelial injury, bile duct damage, and microvascular inflammation, and these changes may promote graft dysfunction (14). Jiang et al. (24) reported that HLA class II antigens were more strongly associated with graft dysfunction than HLA class I antigens. Similar findings have also been reported in liver transplantation. Persistent class II DSA has been linked to poorer graft outcomes and a higher risk of AMR-related injury (20). These findings support an important role for HLA class II alloimmunity in long-term graft injury and in the development of AMR after liver transplantation.

In clinical practice, clinicians often use mean fluorescence intensity (MFI) to assess DSA levels. Higher DSA-MFI values are often associated with a higher risk of AMR-related injury or adverse graft outcomes after liver transplantation (25, 26). Still, high-level DSA does not always lead to clinically overt AMR. At present, there is no universally accepted MFI cutoff for high-risk DSA in liver transplantation. As shown in **Table 1**, the reported thresholds vary across studies. Some studies used relatively high thresholds, such as One Lambda >10, 000 or Immucor >5, 000, whereas others defined strongly positive DSA as MFI ≥10, 000 (15). Other studies used lower thresholds, such as DSA MFI >2, 000, sMFI ≥5, 000, or MFI ≥1, 000 (14, 27, 28). Some studies did not use a fixed cutoff. Instead, they reported absolute MFI values, total DSA burden, or complement-binding activity, such as sum-MFI ≥10, 000 or increased C1q/C3d-binding DSA activity in AMR-associated case (26, 29). Still, the definition of “high-risk” MFI is not consistent across studies. This variation may reflect differences in recipient populations, transplant settings, AMR phenotypes, assay interpretation, and clinical endpoints. For this reason, MFI should not be used alone to judge DSA pathogenicity. It should be interpreted together with antibody persistence, antigen specificity, histopathological findings, and clinical manifestations (17, 30, 31). Therefore, DSA positivity and MFI values should be regarded as risk indicators rather than standalone diagnostic criteria for LT-AMR. Their interpretation requires integration with antibody persistence, antigen specificity, complement-binding capacity, C4d staining, histologic injury, and clinical graft dysfunction.

**Table 1:** Studies reporting DSA-MFI definitions or thresholds relevant to AMR risk in liver transplantation.

Year	Study	Population/design	DSA type	Assay	MFI definition/threshold	AMR cases n	Main finding
2017	Kozlowski et al.	Adult; cohort	pDSA	SAB	≥5, 000	NR	Higher C1q-MFI predicted early severe AMR.
2019	Tamura et al.	Adult; retrospective	pDSA	SAB	≥1, 000	NR	DSA was associated with higher 90-day mortality.
2020	Del Bello et al.	Adult; cohort	pDSA	SAB	Lambda >10, 000; Immucor>5, 000	10	High-MFI pDSA increased acute rejection risk.
2022	Maurice et al.	Adult; biopsy-based	DSA	SAB	>2, 000	NR	MFI >2, 000 was used in aAMR evaluation.
2023	Tajima et al.	Adult; retrospective	pDSA	SAB	≥500; sum-MFI ≥10, 000	5	sum-MFI ≥10, 000 identified high-risk pDSA.
2023	El Hag et al.	Adult; biopsy-based	DSA	SAB	≥5, 000	5	sMFI ≥5, 000 correlated with higher C4d and aAMR.
2023	Ogawa et al.	Adult; single center	pDSA	SAB	≥10, 000	1	AMR occurred in 1 strongly positive DSA case.
2025	Kanaan et al.	Pediatric; retrospective	DSA	SAB	≥1, 000	NR	DSA+ with AMR showed higher HLA-II MFI.

These studies highlight the heterogeneity of reported MFI definitions and the absence of a universally accepted high-risk cutoff.

AMR, antibody-mediated rejection; aAMR, acute antibody-mediated rejection; DSA, donor-specific antibody; HLA-II, HLA class II; LDLT, living donor liver transplantation; MFI, mean fluorescence intensity; NR, not reported; pDSA, preformed donor-specific antibody; SAB, single-antigen bead.

## 4 Effector mechanisms of liver AMR

### 4.1 Complement-dependent and complement-independent injury

When DSA bind to donor HLA class I or class II antigens, they form antigen–antibody complexes. These complexes can bind complement component C1q and start the classical complement cascade. C1q binding activates C1r and C1s in sequence. This process then cleaves C4 and C2 and forms the C3 convertase (C4b2a). The C3 convertase cleaves C3 and produces C3b. It also helps form the C5 convertase (C4b2a3b). The C5 convertase cleaves C5 and produces C5b. C5b then binds C6, C7, C8, and C9 in sequence and forms the membrane attack complex (MAC) on the cell membrane (10). MAC deposition can directly injure endothelial cells and other target cells. This process can contribute to graft damage (32). Small complement fragments such as C3a and C5a also play an important role. These molecules act as strong anaphylatoxins. They recruit neutrophils, macrophages, and other inflammatory cells to the site of injury and further increase local inflammation (33).

Deposition of complement split products such as C4d and C3d in graft tissue is generally seen as evidence of classical complement activation. It also provides supportive immunopathological evidence for the diagnosis of AMR (34). Still, complement activation alone does not fully explain the full range of AMR-related injury in liver transplantation. More evidence now suggests that complement-independent mechanisms also contribute to AMR. In these settings, the Fc portion of antibodies can bind Fcγ receptors on innate immune effector cells, including natural killer (NK) cells, macrophages, and monocytes (35). This interaction can trigger antibody-dependent cellular cytotoxicity (ADCC), increase leukocyte recruitment, and promote endothelial activation (36). These processes can increase graft inflammation even when clear complement-mediated injury is not present (37).

The local inflammatory environment may also increase the susceptibility of the liver graft to AMR. A “two-hit” model has therefore been proposed (38). In this model, an initial graft injury, such as T cell-mediated rejection (TCMR), ischemia–reperfusion injury, or viral hepatitis, increases local inflammation and may upregulate HLA class II expression on graft cells (38, 39). This change may increase the likelihood of subsequent DSA binding, complement activation, and tissue injury. It may also help explain why some recipients with circulating DSA remain clinically stable, whereas others develop overt graft dysfunction and histologically evident rejection (1, 4).

### 4.2 Endothelial activation and microvascular inflammation

In liver AMR, graft endothelium is not only a passive target of circulating DSA (36). It also takes part in the progression of local inflammatory injury. After DSA binding and complement- and/or Fc receptor-mediated effector activation, endothelial cells may become swollen, functionally disturbed, and pro-inflammatory. These changes can promote leukocyte adhesion and transmigration into the graft microvasculature (40, 41). On histopathology, this process is reflected by portal microvascular dilatation, endothelial cell hypertrophy or swelling, capillaritis, and microvasculitis. These changes are among the characteristic features described in acute AMR of the liver allograft (21, 27).

Microvascular inflammation may be a key link between humoral alloimmunity and structural graft injury. Activated endothelium can recruit neutrophils, macrophages, monocytes, and other innate immune effector cells. This process can increase tissue injury beyond the initial antibody-binding event (36, 42, 43). Endothelial injury may also disrupt the microcirculation of the portal tract and the peribiliary vascular plexus. This damage may contribute to ischemia-like bile duct injury, ductopenic change, and progressive graft fibrosis (44, 45). These findings support the idea that AMR in liver transplantation is not defined only by circulating antibodies or complement deposition. It is also defined by downstream endothelial dysfunction and microvascular inflammatory injury (3, 4).

Microvascular inflammation in liver AMR often overlaps with other forms of graft injury. As a result, isolated AMR is uncommon; some DSA-positive recipients remain clinically stable, and AMR in liver transplantation is often under-recognized in routine practice (14). Persistent endothelial activation and unresolved microvascular injury may link acute humoral injury to chronic bile duct loss, ongoing fibrosis, and long-term graft dysfunction (13).

## 5 Cellular network in liver AMR

### 5.1 Endothelial cells

Endothelial cells are central targets of antibody-mediated injury, but they also actively participate in the pathogenesis of AMR. In the liver allograft, DSA bind donor HLA molecules on vascular and microvascular endothelium (43). This interaction causes not only complement deposition and cellular injury, but also endothelial activation. Activated endothelial cells upregulate adhesion molecules (46), alter cytokine and chemokine production, and promote leukocyte adhesion and transmigration, thereby amplifying local inflammation (36). The endothelium should therefore be viewed not only as a passive target of humoral alloimmunity, but also as an active immune interface linking circulating alloantibodies to downstream microvascular injury (46).

In liver transplantation, endothelial injury is closely related to the characteristic histopathologic features of AMR, including portal vein endothelial hypertrophy or swelling, portal microvascular dilatation, capillaritis, and microvasculitis (47). Persistent endothelial activation may also damage the portal microcirculation and the peribiliary vascular plexus (48), contributing to bile duct injury, cholestatic change, and progressive graft fibrosis (49).

### 5.2 Macrophages

Macrophages are major innate immune effector cells with phagocytic, antigen-presenting, and cytotoxic functions. The monocyte–macrophage lineage is now recognized as an important contributor to both acute and chronic allograft injury (36, 50). In AMR, macrophages may participate in both tissue injury and tissue remodeling by responding to antibody-coated targets, complement fragments, and danger-associated molecular patterns released from injured graft cells. Rather than acting as passive bystanders, they can intensify local inflammation and prolong endothelial and parenchymal injury (50, 51).

In experimental liver transplantation, Zimmerer et al. showed that alloantibody-dependent hepatocellular allograft rejection can be mediated by host macrophages through Fcγ receptor signaling and reactive oxygen species (ROS)-dependent cytotoxic mechanisms (42). More recently, Terry et al. showed in human liver transplantation that disulfide-HMGB1 can signal through TLR4 and TLR9 and induce inflammatory macrophages that support innate–adaptive crosstalk (50). Together, these findings suggest that macrophages may act as key amplifiers of humoral injury in liver AMR, particularly when alloantibody-mediated endothelial injury occurs in the setting of ischemia-reperfusion injury or other inflammatory triggers.

### 5.3 Neutrophils and NETs

Neutrophils are among the earliest innate immune cells recruited to sites of graft injury and are increasingly implicated in antibody-mediated tissue damage (52). In solid-organ transplantation, they may contribute to AMR through direct inflammatory infiltration as well as through the formation of neutrophil extracellular traps (NETs) (53). NETs consist of extracellular DNA, histones, and granule proteins, and these structures can promote endothelial injury and amplify local inflammation (53–55). Neutrophil-rich capillaritis and microvascular inflammation are therefore increasingly recognized as part of the broader effector landscape of AMR.

Direct evidence in liver AMR remains limited. However, Liu et al. reported in liver transplantation models and patient samples that NET formation was associated with acute rejection and that NETs promoted Kupffer cell M1 polarization and HMGB1/TLR4/MAPK signaling, thereby aggravating local inflammatory injury (53). These observations support a potential role for neutrophils and NETs in endothelial and microvascular injury in liver AMR, although more liver-specific mechanistic studies are still needed.

### 5.4 NK cells

Natural killer (NK) cells are abundant innate lymphocytes in the liver and have both cytotoxic and immunoregulatory functions (56, 57). Depending on the local immune context, they may support either tolerance or rejection. On the one hand, hepatic NK cells may promote immune tolerance after liver transplantation by limiting alloreactive T-cell responses. On the other hand, in AMR they may function as potent effector cells by recognizing antibody-coated endothelial targets through FcγRIII (CD16) and mediating antibody-dependent cellular cytotoxicity (ADCC) (58, 59).

More evidence from kidney and lung transplantation suggests that NK cells are closely linked to microvascular inflammation in AMR (59, 60). In liver transplantation, hepatic NK cells have been reported to play dual roles in both rejection and tolerance (61, 62). In lung transplantation, Calabrese et al. showed that CD16+ NK cells were associated with antibody-mediated rejection and chronic allograft dysfunction (63). These findings support the possibility that NK cells may connect circulating DSA to endothelial injury in liver AMR, particularly through CD16-dependent pathways, although direct liver-specific evidence remains limited (35, 64).

### 5.5 B cells, plasma cells, and T-cell help

B cells and plasma cells are the main cellular sources of DSA and therefore occupy a central position in the humoral immune network of AMR (17). After donor antigen exposure, activated B cells can differentiate into plasmablasts, antibody-secreting plasma cells, and memory B cells (65). This process supports both immediate and long-term humoral responses. In liver transplantation, more evidence now shows that DSA-producing B-cell responses are linked to poor graft outcomes (1). These outcomes include acute rejection, chronic rejection, ductopenic injury, fibrosis, and plasma cell-rich rejection (PCRR). Still, the strength and timing of these associations may differ across clinical settings (45).

Plasma cells are especially important in persistent or treatment-refractory humoral injury (66). Long-lived plasma cells may continue to produce alloantibodies even after conventional immunosuppression or temporary antibody-depleting treatment, whereas memory B cells may serve as a reservoir for renewed antibody production after re-exposure to donor antigen (65). Most mechanistic evidence regarding long-lived plasma cells and memory B cells comes from kidney transplantation and broader AMR research (67). Even so, these concepts are highly relevant to liver transplantation, where persistent class II DSA and recurrent humoral injury remain important clinical problems (68).

Liver-specific findings also support the importance of B-cell- and plasma cell-driven alloimmunity. O’Leary et al. showed that donor-specific HLA antibodies in liver transplantation are linked not only to acute AMR, but also to ductopenia, biliary injury, fibrosis, and plasma cell hepatitis-like lesions (6, 45). More recently, Ozturk et al. reported that in post-liver transplant plasma cell-rich rejection, DSA was detected in 16 of 19 tested patients and C4d was positive in 9 of 10 cases (69). These findings support the possibility that at least some PCRR lesions fall within the histologic spectrum of humoral rejection and may reflect sustained antibody-driven immune injury rather than a purely T cell-mediated process (70).

CD4+ T-cells strongly shape the B-cell response after transplantation. T follicular helper (Tfh) cells are especially important in this process (71). Tfh cells promote germinal center formation, B-cell maturation, class switching, and differentiation into plasmablasts and plasma cells (71, 72), with IL-21 and costimulatory pathways serving central roles in this process (72). In transplantation more broadly, Tfh cells are now increasingly linked to donor-specific alloantibody generation and graft rejection (73).

Liver-specific data also support a role for Tfh-related biology in post-transplant humoral immunity (74). Zhang et al. showed that circulating Tfh cells in liver transplant recipients could help B cells differentiate into plasmablasts in an IL-21-dependent manner (75). This finding suggests that cTfh cells may take part in alloreactive responses after liver transplantation. Ono et al. also reported that polymorphisms in Tfh-related genes, including BCL6 and IL-21, were associated with *de novo* DSA formation after living-donor liver transplantation (72). Although these findings do not establish a direct Tfh-driven mechanism of liver AMR, they strongly support T-cell help as an upstream regulator of DSA persistence and pathogenicity.

## 6 Downstream inflammatory pathways in LT-AMR: evidence strength and mechanistic extrapolation

The cellular effectors described above are connected through several downstream inflammatory and regulatory pathways that may amplify or sustain AMR-related graft injury. However, the strength of the evidence varies widely across pathways. In this section, we classify the evidence into three practical categories: (i) pathways supported by liver-specific AMR studies, (ii) pathways supported by liver transplantation rejection models or liver allograft inflammation studies but not specifically by LT-AMR data, and (iii) pathways extrapolated mainly from kidney, heart, lung transplantation, or *in vitro* endothelial models. This evidence-based separation is important because several inflammatory pathways may reflect a permissive or amplifying graft environment rather than a defining mechanism of LT-AMR itself. (**Table 2**).

**Table 2:** Potential inflammatory and regulatory pathways relevant to LT-AMR or AMR-associated graft injury: evidence source and interpretation.

Year	Author	Pathway	Model	Interpretation for LT-AMR	Main finding
2025	Huang et al.	NF-κB	Animal/Cell	Indirect inflammatory amplifier; not LT-AMR-specific.	ANGPTL4 alleviated rejection by inhibiting NF-κB signaling and promoting Kupffer cell M2 polarization.
2025	Qin et al.	PI3K/Akt/mTOR	Animal/Cell	Indirect macrophage-regulatory pathway; not AMR-specific.	LXRα agonist alleviated acute rejection after liver transplantation by regulating macrophage polarization through ABCA1/MAPK and PI3K/AKT/mTOR signaling.
2024	Awad et al.	IL-6-related susceptibility	Human	Indirect clinical evidence; limited LT-AMR validation.	IL-6 polymorphism and circulating inflammatory cytokines were associated with early rejection after living donor liver transplantation.
2024	Ono et al.	Tfh-related genes (BCL6/IL-21)	Human	Upstream DSA-related evidence, not direct tissue injury.	Polymorphisms in BCL6 and IL-21 were associated with *de novo* DSA formation after liver transplantation.
2023	Tajima et al.	Complement/C5 axis	Animal	Direct liver-specific AMR evidence; strong therapeutic target.	Anti-C5 treatment reduced graft injury, decreased C4d burden, and improved survival in a rat LT-AMR model.
2022	Liu et al.	HMGB1/TLR4/MAPK	Animal/Cell	Indirect inflammatory pathway; not LT-AMR-specific.	NETs promoted HMGB1 translocation and Kupffer cell M1 polarization, supporting HMGB1/TLR4/MAPK-related inflammatory amplification in acute liver transplant rejection.
2022	Mang et al.	IL-6/JAK/STAT3	Animal	Liver-specific AMR-like evidence; needs clinical validation.	In a pre-sensitized rat OLT model, high DSA, C4d deposition, and IL-6/JAK1/STAT3 upregulation were observed in AMR-like injury.
2021	Tang et al.	NFAT-BATF/JUN/IRF4-IL-21	Animal/Cell	Indirect upstream Tfh evidence; AMR role uncertain.	Tacrolimus suppressed Tfh-related transcriptional signaling and IL-21, supporting an upstream Tfh regulatory axis in liver transplant rejection.
2018	Zhang et al.	IL-22/STAT3	Animal	Indirect STAT3-related inflammation; not AMR-specific.	IL-22 showed a dual effect, appearing protective during ischemia-reperfusion injury but pro-inflammatory during acute rejection with increased p-STAT3 and altered Th17/Treg balance.
2018	Zhao et al.	PI3K/Akt	Animal/Cell	Indirect macrophage-polarization evidence.	IL-34 induced Kupffer cell M2 polarization through PI3K/Akt and attenuated acute rejection after liver transplantation.
2018	Jin et al.	Src/FAK/PI3K/Akt/ERK/mTOR	Cell	Extrapolated endothelial mechanism; not liver specific.	HLA class II antibody ligation directly activated endothelial signaling and promoted endothelial proliferation/migration.
2018	Zhang et al.	Tfh-IL-21-B-cell axis	Human	Upstream DSA-regulatory pathway, not effector injury.	Circulating Tfh cells promoted IL-21-dependent B-cell differentiation, supporting an upstream role in humoral alloimmunity.

The final column distinguishes direct liver-specific AMR evidence from indirect, extrapolated, or hypothesis-generating mechanisms.

### 6.1 IL-6/JAK/STAT3 signaling

Interleukin-6 (IL-6) is a pleiotropic cytokine involved in liver regeneration, inflammatory regulation, and alloimmune responses after transplantation (56, 76). In liver transplantation, elevated IL-6 levels and IL-6-related genetic polymorphisms have been associated with acute rejection (77), supporting a role for IL-6 signaling in graft inflammatory injury. In a pre-sensitized rat model of liver transplantation-associated AMR, Mang et al. reported increased expression of IL-6, JAK1, and STAT3 (78). Awad et al. also found that IL-6 polymorphisms and circulating inflammatory cytokines were associated with early rejection after living donor liver transplantation (77). Even so, liver-specific evidence remains limited, and much of the current support derives from acute rejection studies or experimental models rather than direct clinical studies of liver AMR.

IL-22/STAT3 signaling may interact with this inflammatory network. In a rat allogeneic liver transplantation model, Zhang et al. showed that IL-22 exerted a dual effect: it appeared protective during ischemia-reperfusion injury but promoted inflammation during acute rejection through changes in the Th17/Treg balance and STAT3-related signaling (79). This pathway is therefore more strongly linked to graft inflammation and immune remodeling than to liver AMR itself. Thus, IL-6/JAK/STAT3 signaling should be interpreted as a plausible inflammatory amplification pathway in LT-AMR, supported by liver-specific experimental data but still lacking sufficient validation in large clinical LT-AMR cohorts.

### 6.2 PI3K/Akt/mTOR signaling

The PI3K/Akt/mTOR pathway regulates cell survival, metabolism, proliferation, and immune cell differentiation. In AMR biology, anti-HLA antibodies can directly activate PI3K/Akt/mTOR-related signaling in endothelial cells (80), suggesting that antibody ligation may convert endothelium from a passive target into an active contributor to graft inflammation (40). Guo et al. further showed that HLA class II antibodies activated PI3K/Akt/mTOR signaling in endothelial cells and promoted PBMC migration and differentiation toward CD68^+^/CD163^+^ M2-like macrophages (41). These findings suggest that antibody-triggered endothelial signaling may also shape downstream myeloid responses.

In liver transplantation, this pathway may have context-dependent effects. Zhao et al. showed that IL-34 induced Kupffer cell M2 polarization through PI3K/Akt signaling and attenuated acute rejection in a rat liver transplantation model (81). Qin et al. further reported that LXRα agonists improved acute rejection through PI3K/Akt/mTOR-related regulation of macrophage polarization (82). Therefore, the PI3K/Akt/mTOR pathway is best considered an extrapolated endothelial and macrophage-regulatory mechanism that may be relevant to LT-AMR, rather than a pathway already established as liver AMR-specific.

### 6.3 NF-κB signaling

NF-κB is a central inflammatory signaling pathway involved in cytokine production, innate immune activation, and tissue injury. In liver transplantation, NF-κB activation has been linked more clearly to acute rejection-related inflammatory damage than to AMR alone (83). Huang et al. showed in a rat orthotopic liver transplantation model that ANGPTL4 reduced acute rejection by inhibiting NF-κB signaling and promoting Kupffer cell M2 polarization (83). NF-κB is therefore best viewed as a broad inflammatory pathway relevant to graft injury, including antibody-associated injury, rather than a liver AMR-specific signature pathway (84, 85). Accordingly, NF-κB activation should be regarded as a general inflammatory amplifier in liver allograft injury, with potential relevance to AMR but limited specificity for LT-AMR.

### 6.4 HMGB1/TLR4/MAPK signaling

HMGB1 is a damage-associated molecular pattern molecule that can activate innate immune responses through receptors such as TLR4 (50). In liver transplantation, Liu et al. showed that NETs promoted HMGB1 translocation and Kupffer cell M1 polarization (53). NET-associated signaling through TLR4/MAPK then further increased local inflammatory injury during acute rejection. These findings support the idea that the HMGB1/TLR4/MAPK axis may act as a pro-inflammatory amplification loop in liver graft injury (53). However, current evidence more strongly supports a role in acute rejection and ischemia-reperfusion-related inflammation than in liver AMR specifically (84). Therefore, the HMGB1/TLR4/MAPK axis is better interpreted as part of a permissive inflammatory graft environment that may lower the threshold for antibody-mediated injury, rather than as a validated driver of LT-AMR.

### 6.5 Complement/C5a–C5b-9 amplification axis

Complement activation has already been discussed as a core effector mechanism in liver AMR (4). The downstream C5a–C5b-9 axis deserves separate attention because it links antibody binding to both inflammatory cell recruitment and direct tissue injury. Cleavage of C5 produces C5a and C5b. C5a is a potent anaphylatoxin that promotes leukocyte recruitment and inflammatory amplification, whereas C5b contributes to membrane attack complex formation (86). In a rat model designed to meet Banff criteria for liver transplantation AMR, Tajima et al. showed that anti-C5 antibody treatment improved AMR (87). This provides direct liver-specific evidence that terminal complement activation is mechanistically important in LT-AMR and identifies the C5 axis as one of the strongest candidate therapeutic targets in this field (87). Among the pathways discussed in this review, the C5 axis currently represents one of the strongest liver-specific mechanistic and therapeutic candidates for LT-AMR.

### 6.6 Endothelial signaling downstream of antibody ligation

Donor-specific HLA antibodies can do more than trigger complement deposition; they can also directly activate intracellular signaling in endothelial cells (36, 43). Experimental studies have shown that HLA class I and class II antibody ligation can activate signaling networks that involve Src family kinases, focal adhesion kinase, ERK, PI3K/Akt, and mTOR. These signals can lead to cytoskeletal rearrangement, cell survival, adhesion molecule expression, chemokine production, and increased leukocyte adhesion (40). Although these mechanisms are not liver-specific, they provide a plausible explanation for the endothelial activation and microvascular inflammation observed in liver AMR (46). They also help explain how alloantibody binding can intensify local graft injury beyond complement deposition alone (80).

### 6.7 Tfh–IL-21–B-cell axis

Whereas the pathways discussed above mainly reflect downstream inflammatory amplification, the T follicular helper (Tfh)–IL-21–B-cell axis is more closely related to the generation and persistence of DSA (88). Tfh cells support germinal center responses and drive B-cell maturation, class switching, and differentiation into plasmablasts and plasma cells, with IL-21 serving as a central mediator in this process (89). In liver transplantation, Zhang et al. showed that circulating Tfh cells from liver transplant recipients could promote B-cell differentiation into plasmablasts in an IL-21-dependent manner, suggesting that cTfh cells participate in post-transplant alloreactive humoral responses (73). Ono et al. further reported that polymorphisms in Tfh-related genes, including BCL6 and IL-21, were associated with *de novo* DSA formation after liver transplantation (72). Together, these observations place the Tfh–IL-21–B-cell axis within the upstream immune network that shapes humoral alloimmunity in LT. Thus, the Tfh–IL-21–B-cell axis is better positioned as an upstream regulator of DSA generation and persistence, rather than as a direct effector pathway of graft injury in LT-AMR. (**Table 2**).

## 7 Clinicopathologic manifestations and diagnostic challenges

### 7.1 Acute and chronic antibody-mediated rejection

Acute antibody-mediated rejection (aAMR) refers to early antibody-mediated injury of the liver allograft and usually develops within days to weeks after transplantation in the presence of circulating DSA (14). According to the Banff framework, the diagnosis should be considered when compatible histopathologic findings are accompanied by circulating DSA and graft C4d deposition after exclusion of other causes of graft dysfunction (3). Typical features include portal microvascular dilatation, endothelial cell swelling, capillaritis, and vasculitis (3). Clinically, aAMR is usually associated with graft dysfunction and elevated serum transaminases, and some patients may also develop thrombocytopenia (1). Diagnosis remains difficult because the clinical presentation is often nonspecific, subclinical immune abnormalities may be present, and aAMR may coexist with T cell-mediated rejection (TCMR). As a result, aAMR is likely underdiagnosed, and its true incidence after liver transplantation remains uncertain (1).

Chronic antibody-mediated rejection (cAMR) is a progressive form of graft injury that develops months to years after transplantation and is associated with sustained humoral alloimmunity and persistent DSA (45). The Banff Working Group describes cAMR as a clinicopathologic entity characterized by portal or periportal and perivenular plasma cell-rich inflammation, interface activity, and progressive noninflammatory fibrosis, with diagnosis supported by circulating DSA and focal C4d deposition after exclusion of competing causes of injury (3). Persistent donor-specific alloantibodies have also been linked to chronic bile duct injury, ductopenia-like change, and progressive graft fibrosis, which may contribute to long-term graft dysfunction (45, 90). Recognition is often delayed because liver function can remain near normal in early disease and protocol biopsies are not routinely performed in many centers (49).

### 7.2 Overlap with TCMR and PCRR

In clinical practice, aAMR and TCMR frequently overlap rather than appearing as separate entities (2, 3). Portal inflammation, bile duct injury, endothelial activation, and graft dysfunction may be shared across rejection phenotypes (91). Therefore, distinction between aAMR, TCMR, and plasma cell-rich rejection requires integrated assessment of histology, DSA, C4d staining, graft biochemistry, timing after transplantation, treatment response, and exclusion of competing causes of graft injury (2).

A similar diagnostic problem arises with plasma cell-rich rejection (PCRR), also called plasma cell-rich hepatitis-like rejection (69). PCRR is an uncommon but clinically important form of liver allograft rejection characterized by marked portal and/or perivenular plasma cell infiltration, often exceeding 30% of the inflammatory infiltrate (70). Its relationship with AMR remains unsettled. Current evidence suggests that PCRR is heterogeneous and may combine features of TCMR, humoral immune activation, and autoimmune-like injury rather than representing a uniform subtype of AMR (92). Associations with DSA, C4d positivity, and poor graft outcomes suggest that at least some cases lie within the broader spectrum of humoral rejection, although its exact classification and pathogenesis remain unresolved (70). To clarify the diagnostic distinction among overlapping rejection phenotypes, **Table 3** summarizes the main clinicopathologic differences among aAMR, cAMR, TCMR, and PCRR.

**Table 3:** Clinicopathologic comparison of aAMR, cAMR, TCMR, and PCRR after liver transplantation.

Feature	aAMR	cAMR	TCMR	PCRR
Main mechanism	DSA-associated humoral injury	Persistent humoral alloimmunity with chronic graft injury	T cell-driven alloimmune injury	Plasma cell-rich immune injury with mixed cellular, autoimmune-like, or humoral features
Main target	Portal microvasculature and graft endothelium	Portal/periportal and perivenular regions, bile ducts, and microvasculature	Portal tracts, bile ducts, and venous endothelium	Portal and/or perivenular areas
Histologic pattern	Portal microvascular dilatation, endothelial swelling, capillaritis/microvasculitis, portal edema	Fibrosis, bile duct injury, ductopenic change, chronic graft dysfunction	Portal inflammation, bile duct injury, portal or central venous endotheliitis	Prominent plasma-cell infiltrates, interface activity, hepatitic or perivenular injury
Laboratory clues	Circulating DSA; C4d supports diagnosis; MFI/complement-binding may aid risk assessment	Persistent or *de novo* DSA, often class II; C4d may support diagnosis	No DSA requirement; diagnosis mainly biopsy-based	Plasma cell-rich biopsy; DSA/C4d may be present in some cases
Treatment focus	Optimize immunosuppression; PE/IVIG; selected rituximab, bortezomib, or complement-directed therapy	Individualized immunosuppression; anti-humoral therapy in active cases; retransplantation if irreversible	Corticosteroids and optimization of immunosuppression; lymphocyte-depleting therapy if steroid-resistant	Optimize immunosuppression and corticosteroids; consider anti-humoral therapy if DSA/C4d-positive

Overlap among these phenotypes is common; final diagnosis requires integrated assessment of timing, graft dysfunction, histology, DSA, C4d staining, and exclusion of competing causes.

### 7.3 Limitations of current diagnostic criteria

**Figure 1:**
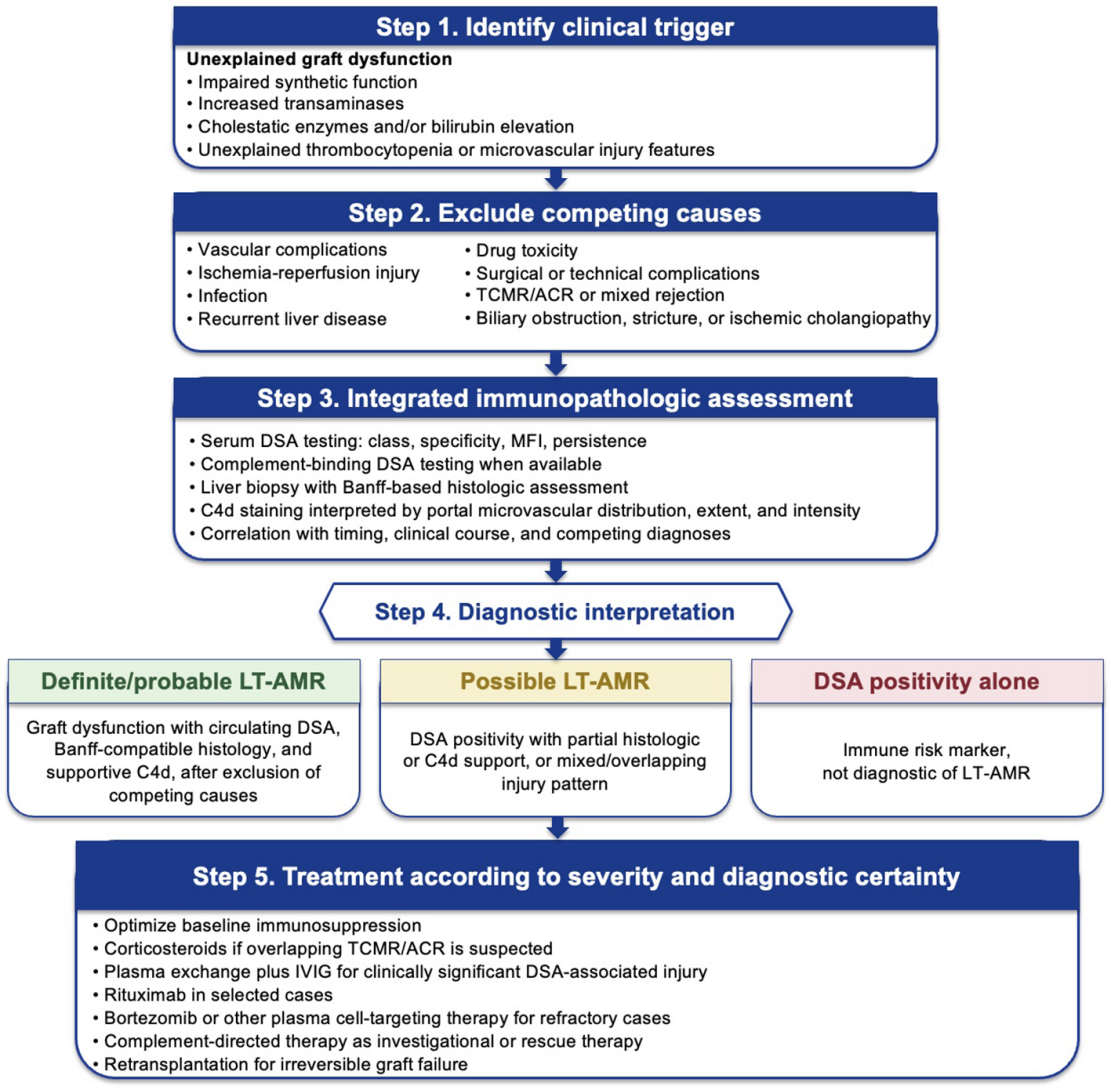
Practical diagnostic-therapeutic algorithm for suspected LT-AMR.

The Banff Working Group criteria remain the most widely used framework for diagnosing liver allograft AMR, but their application in routine practice is still challenging (3). In one interinstitutional study, only a small proportion of DSA-positive liver transplant recipients met the full criteria for acute AMR, whereas most suspected cases showed overlapping features with TCMR rather than isolated AMR (14). Some patients display only subclinical immunologic abnormalities or nonspecific histopathologic changes that do not meet current diagnostic thresholds. These limitations likely contribute to under-recognition of AMR after liver transplantation and highlight the need for more sensitive and biologically informed diagnostic approaches (92). To translate these diagnostic considerations into clinical practice, **Figure 1** summarizes a practical diagnostic-therapeutic algorithm for suspected LT-AMR by integrating graft dysfunction, exclusion of competing causes, DSA and complement-binding assessment, Banff-based histologic evaluation, C4d staining, diagnostic stratification, and stepwise treatment selection.

This algorithm integrates clinical graft dysfunction, exclusion of competing causes, DSA assessment, complement-binding testing, Banff-based histology, C4d staining, and diagnostic stratification for suspected LT-AMR. Treatment is guided by diagnostic certainty and injury severity and may include optimization of baseline immunosuppression, corticosteroids for overlapping TCMR/ACR, plasma exchange plus IVIG, rituximab, plasma cell-targeting therapy, investigational complement-directed therapy, or retransplantation for irreversible graft failure.

## 8 Mechanism-based therapeutic strategies for liver AMR

To connect the treatment strategies discussed below with the underlying pathogenic components and clinicopathologic findings, **Table 4** summarizes the major mechanism–pathology–treatment links in LT-AMR.

**Table 4:** Mechanism–pathology–treatment links in LT-AMR.

Pathogenic component	Key mechanism	Key findings	Therapeutic implication
DSA binding	Donor HLA recognition; possible non-HLA antibodies	Risk DSA profiles	DSA monitoring; optimize immunosuppression
Complement-mediated injury	C1q–C4d–C3/C5–MAC activation	C4d deposition; endothelial injury	PE/IVIG; complement blockade investigational
Fc receptor-mediated injury	Fc engagement of innate immune cells	Leukocyte recruitment; endothelial injury	Anti-humoral therapy; future innate-targeted strategies
Endothelial activation	Antibody/complement/Fc-related activation	Endothelial swelling; microvascular dilatation	Integrate biopsy, DSA, C4d, graft function
Microvascular inflammation	Inflammatory cell recruitment	Capillaritis/microvasculitis; possible TCMR overlap	Evaluate mixed rejection; steroids if TCMR overlaps
B-cell/plasma-cell response	DSA generation and persistence	Persistent DSA; refractory injury	Rituximab; bortezomib/plasma cell-targeted therapy
Chronic humoral injury	Persistent alloantibody-mediated graft injury	Fibrosis; bile duct injury; ductopenia; chronic graft dysfunction	Longitudinal DSA/biopsy monitoring; retransplantation if irreversible

Therapeutic implications are practical considerations rather than standardized treatment recommendations.

### 8.1 Optimization of baseline immunosuppression

There is still no universally accepted treatment algorithm for AMR after liver transplantation (91). In clinical practice, treatment should be individualized according to diagnostic certainty, severity of graft dysfunction, DSA profile, C4d deposition, Banff-compatible histologic injury, and the presence of overlapping TCMR or PCRR (92). Therefore, therapy is best considered as a stepwise escalation strategy, beginning with optimization of baseline immunosuppression and treatment of concurrent cellular rejection (93), followed by anti-humoral or targeted approaches when clinically significant DSA-associated injury is suspected or confirmed (94). Calcineurin inhibitors, mycophenolate mofetil, and corticosteroids remain the main components of routine post-transplant immunosuppression (93). Although these agents are not specific treatments for AMR, they provide background control of alloimmune activation while anti-humoral therapies are introduced when AMR is suspected or confirmed (95).

### 8.2 Antibody removal and neutralization: plasmapheresis and IVIG

Because circulating DSA is central to the pathogenesis of AMR, plasmapheresis, or plasma exchange (PE), and intravenous immunoglobulin (IVIG) are among the most commonly used first-line anti-humoral therapies (91). PE reduces the circulating alloantibody burden, whereas IVIG may neutralize alloantibodies, modulate B-cell responses, and attenuate complement-mediated injury (96). In reported cases of liver transplant AMR, PE and IVIG are usually given in combination with corticosteroids and intensified maintenance immunosuppression rather than as isolated interventions (7). Their role is particularly well established in ABO-incompatible liver transplantation, where antibody-removal strategies have clearly improved outcomes (97). Even so, these approaches may be insufficient in patients with persistent high-level DSA or ongoing plasma cell-driven antibody production (95).

### 8.3 B-cell targeting: rituximab

Rituximab, an anti-CD20 monoclonal antibody, depletes circulating B cells and may help limit *de novo* antibody production (98). In liver transplantation, it is widely used in desensitization protocols for ABO-incompatible transplantation (99), and has also been incorporated into treatment regimens for suspected or biopsy-proven AMR, usually alongside PE and IVIG (98). Its role in LT-AMR appears to be greatest as part of combination therapy rather than as monotherapy (100). However, rituximab does not directly eliminate long-lived plasma cells, which may limit its efficacy in persistent humoral injury. Infection risk is also an important concern, particularly when rituximab is combined with other lymphocyte-depleting therapies (101).

### 8.4 Plasma cell targeting: bortezomib

Persistent alloantibody production is largely sustained by plasma cells, making proteasome inhibition a potential rescue strategy in refractory AMR (101). Bortezomib has shown activity in kidney transplantation and has also been reported to be beneficial in selected cases of liver transplant AMR, particularly after failure of steroids, PE, IVIG, and rituximab (102, 103). In the best-described liver transplant cases, bortezomib treatment was followed by rapid improvement in graft dysfunction and histologic injury (103). Nevertheless, the available evidence remains limited to case reports and small case series, and the optimal timing, dosing, and safety profile of bortezomib in LT-AMR remain uncertain (91).

### 8.5 Complement-targeted strategies

Complement inhibition is one of the most attractive mechanism-based approaches to LT-AMR (87). Terminal complement activation links DSA binding to both inflammatory amplification and membrane attack complex-mediated tissue injury. Because of this, the C5 axis has emerged as a promising therapeutic target (32). In a rat model designed to satisfy Banff criteria for liver transplant AMR, anti-C5 antibody treatment significantly improved AMR. This study provides direct liver-specific preclinical support for complement blockade (87). Clinical experience in human LT-AMR is still very limited. Even so, these findings suggest that complement-targeted therapy may become an important future option, especially in patients with strong evidence of complement-fixing DSA and severe microvascular injury (92).

### 8.6 Retransplantation as rescue therapy

For patients who progress to irreversible graft failure despite intensive anti-humoral and supportive treatment, retransplantation remains the only definitive rescue option (104). Its use, however, is constrained by organ shortage, substantial operative risk, and outcomes that are generally inferior to those of primary transplantation (104). In this setting, preoperative evaluation of DSA status and humoral sensitization may help refine risk assessment and inform post-transplant management (105). Even so, the need for retransplantation underscores the importance of early recognition and timely treatment of AMR before irreversible graft injury develops (106).

### 8.7 Potential targeted therapies under investigation

A number of mechanism-based therapies are being explored for liver AMR, although none can yet be considered established treatment (92). These include IL-6 or IL-6 receptor blockade, JAK inhibition, IgG-degrading enzymes such as imlifidase, and agents targeting endothelial or innate immune pathways. Imlifidase is a cysteine protease derived from Streptococcus pyogenes that rapidly cleaves circulating IgG at the lower hinge region, thereby reducing intact antibody-mediated complement activation and Fc receptor-dependent effector functions (107). At present, however, most of the supporting evidence comes from kidney transplantation, other solid-organ AMR settings, or experimental models rather than liver-specific clinical studies (108). These approaches should therefore be regarded as investigational and hypothesis-generating, but they may provide a basis for more targeted treatment strategies in LT-AMR.

## 9 Current controversies and future directions

Several important questions remain unresolved in LT-AMR. Future studies should move beyond DSA positivity or MFI thresholds alone and define biologically meaningful serologic risk profiles that include antibody persistence, class specificity, complement-binding activity, and temporal association with graft dysfunction.

The diagnostic boundaries of cAMR also require further refinement, particularly in patients with overlapping bile duct injury, fibrosis, plasma cell-rich inflammation, recurrent disease, drug toxicity, ischemic injury, or chronic T cell-mediated injury.

Mechanistic studies should prioritize pathways with direct relevance to LT-AMR and distinguish true drivers of antibody-mediated injury from secondary inflammatory responses associated with broader graft injury.

Future biomarker development should integrate serology, C4d staining, histopathology, endothelial and microvascular injury patterns, molecular profiling, and clinical graft dysfunction rather than relying on any single marker.

More precise monitoring strategies are also needed. Rather than relying on routine DSA surveillance alone, future approaches should integrate DSA dynamics, biopsy findings, molecular classifiers, graft biochemistry, and clinical context to guide risk-adapted immunosuppression. Computational models may eventually help combine these variables for earlier AMR recognition, prognostic assessment, and individualized treatment selection, although such tools remain at an early stage in liver transplantation.
